# Advancing precision public health using human genomics: examples from the field and future research opportunities

**DOI:** 10.1186/s13073-021-00911-0

**Published:** 2021-06-01

**Authors:** Megan C. Roberts, Alison E. Fohner, Latrice Landry, Dana Lee Olstad, Amelia K. Smit, Erin Turbitt, Caitlin G. Allen

**Affiliations:** 1 Division of Pharmaceutical Outcomes and Policy, University of North Carolina Eshelman School of Pharmacy, 301 Pharmacy Lane, Chapel Hill, NC 27599 USA; 2 Department of Epidemiology and Institute of Public Health Genetics, University of Washington, 1959 NE Pacific Ave, Seattle, WA 98195 USA; 3 Harvard Medical School, Harvard T.H. Chan School of Public Health, Brigham and Women’s Hospital &The Division of Population Sciences in Dana Farber Cancer Institute, 450 Brookline Ave, Boston, MA 02215-5450 USA; 4 Department of Community Health Sciences, Cumming School of Medicine, University of Calgary, 3280 Hospital Drive NW, Calgary, AB T2N 4Z6 Canada; 5 Cancer Epidemiology and Prevention Research, Sydney School of Public Health, Faculty of Medicine and Health, The University of Sydney, 119-143 Missenden Road, Camperdown, NSW 2050 Australia; 6 Discipline of Genetic Counselling, The University of Technology Sydney, 100 Broadway, Ultimo, NSW 2008 Australia; 7 Department of Behavioral Social and Health Education Sciences, Rollins School of Public Health, Emory University, 1518 Clifton Road, Atlanta, GA 30322 USA

**Keywords:** Precision medicine, Public health, Precision public health, Transdisciplinary research, Human genomics research

## Abstract

Precision public health is a relatively new field that integrates components of precision medicine, such as human genomics research, with public health concepts to help improve population health. Despite interest in advancing precision public health initiatives using human genomics research, current and future opportunities in this emerging field remain largely undescribed. To that end, we provide examples of promising opportunities and current applications of genomics research within precision public health and outline future directions within five major domains of public health: biostatistics, environmental health, epidemiology, health policy and health services, and social and behavioral science. To further extend applications of genomics within precision public health research, three key cross-cutting challenges will need to be addressed: developing policies that implement precision public health initiatives at multiple levels, improving data integration and developing more rigorous methodologies, and incorporating initiatives that address health equity. Realizing the potential to better integrate human genomics within precision public health will require transdisciplinary efforts that leverage the strengths of both precision medicine and public health.

## Background

Precision medicine promises to revolutionize health by incorporating individual-level characteristics (genetic, lifestyle, environmental factors) into medical treatment and prevention strategies to provide “the right care to the right *individual* at the right time” [1]. This approach allows for the application of individually tailored preventive or therapeutic medical interventions that may be more effective than “one size fits all” approaches, thereby improving quality of care and reducing unnecessary diagnostic testing and therapies. It has been debated whether precision-based approaches should be applied not only to individual-level clinical care, but also to efforts to improve health at a population level, i.e., precision public health [2–4]. While the relative merits of targeted versus universal approaches to disease prevention, treatment, and control at the population level remain the subject of debate, there is an increasing recognition that more precise interventions can advance health at a population level [5].

Precision public health is an approach that integrates precision and population-based strategies to provide “the right *intervention* to the right *population* at the right time” [6]. This can include the scaling of precision medicine initiatives and the translation of basic-science discoveries to the population level. A key aspect of precision public health lies in its potential to develop more accurate methods to identify multi-level risk factors and their impact on population health and to use this information to develop targeted policies and programs to promote health [7]. While other -omics approaches (e.g., transcriptomics, proteomics, and metabolomics) are certainly relevant to precision public health, incorporation of human genomics into precision public health initiatives is particularly promising given evidence of substantial heritability of chronic diseases including obesity, cardiovascular disease, and cancer, which are among the major public health challenges of the twenty-first century [8, 9]. Thus, the use of human genomics in precision public health can improve measurement of individual-level factors that shape health, serving as a conduit between precision medicine and public health for improving health across individuals and populations [7, 10].

To date, integrating human genomics research within precision public health initiatives has supported more precise disease prevention and control strategies, including newborn screening and risk-stratified prevention (e.g., enhanced cancer screening for individuals with pathogenic *BRCA1/2* variants) [11–13]. However, currently, application of human genomic research to the translation of precision public health has been relatively limited [14, 15]. Each of the major domains of public health—biostatistics, environmental health, epidemiology, health policy and services, and social and behavioral health—offers complementary opportunities to improve precision public health applications through human genomics [16]. Here, we describe examples of initiatives that integrate human genomics within precision public health research, as well as opportunities to advance research in each of these public health domains. Finally, we discuss challenges and opportunities to integrating human genomics within the field of precision public health that cut across the five major public health domains.

## Examples of integrating human genomics research and public health

We describe ways by which the five domains of public health endorsed by the Association of Schools and Programs of Public Health have integrated consideration of human genomics within precision public health research. The five domains include as follows: biostatistics, environmental health, epidemiology, health policy and health services research, and social and behavioral health (Table 1) [16].

**Table 1:** Illustrative examples of study domains proposed for human genomics research in precision public health

Study domain and definition	Promising opportunities	Specific examples of application in precision public health	Possible future directions	Cross-cutting considerations in health policy, data integration, and health equity
**Biostatistics:** Study of theories and techniques for collecting, analyzing, and interpreting quantitative data relevant to public health issues.	Allow for development of new methods for interrogating complexity of precision public health data	High-throughput genomics to provide more precise modeling and higher resolution of statistical interactions in relation to common or multifactorial complex diseases (e.g., Genomes Project, Genetic Analysis Workshop, ASK2ME) [17].	Development of algorithms for decision support tools and data resources using AI for enhanced resolution and interpretation of multi-omics datasets	• **Data Integration:** addressing data storage and security issues, data integrity, data integration with variables across levels; methods for data analysis. • **Health Equity:** developing novel methods to examine how dimensions of disadvantage interact to create health risks in more precisely defined marginalized subgroups of the population (i.e., according to multiple indicators of social position).
**Environmental Health:** Study of assessment, control, and prevention of factors in the environment that can adversely affect the health of present and future generations.	Elucidate how individual and macro-level factors interact to influence health	Parkinson’s Genes and Environment Study collected mobile health technology for both environmental and personal health information to assess link between pesticide exposure and Parkinson’s disease [20, 21].	Better understanding of mechanisms behind complex environmental exposures and interactions with individual-level factors to target high-risk populations	• **Health Policy:** harmonizing health policies across international and community boundaries to ensure uptake of environmental initiatives that support precision public health. • **Health Equity:** conduct subgroup analyses to examine how different dimensions of social position interact to shape vulnerability to environmental risk factors for poor health.
**Epidemiology:** Application of the scientific method to the study of disease in populations for the purpose of prevention and control.	Improve knowledge of multi-level risk factors to enhance risk assessment and enable risk-stratified screening, treatment, and surveillance	Risk-stratified screening approaches that incorporate genetic risk to improve cost-effectiveness, reduce over diagnosis and maintain the benefits of screening for those at highest risk of breast cancer. Partnering with populations historically absent from biomedical research to improve diversity and equity of epidemiological knowledge (Northwest-Alaska Pharmacogenetics Research Network) [22]	Advancement of our understanding of multi-level risk factors for targeting prevention and screening strategies to populations at highest risk	• **Data Integration:** supporting data sovereignty. • **Health Equity:** ensuring data are collected from diverse, representative groups.
**Health Policy and Services Research**: Research on the cost, access, and quality of the health care system, and on policy issues affecting the organization, financing, and delivery of health care services.	Understanding economic and health impact, as well as health equity of precision public health approaches	Implementing Genomics in Practice Consortium; understanding barriers to implementation of cascade screening for hereditary cancer conditions, such as Lynch syndrome [23].	Evaluation of clinical utility, cost-effectiveness, and patient-reported outcomes	• **Health Policy:** ensuring privacy of potentially highly identifiable information. Considerations of when and how to intervene on genetic information. • **Data Integration:** incorporating genetic test results into electronic health records. • **Health Equity:** integrate consideration of the social determinants of health within health care (e.g., social prescribing).
**Social and Behavioral Science:** Interdisciplinary study focusing on how health education can affect behavior and lifestyle decisions that have an impact on public health.	Translate genomics applications for population health and community-based human genomic research	Assess individuals’ preferences to learn genomic information, assess family-based interventions, implementation of screening approaches, and assess how social environmental inform understanding of gene-environment associations. Efforts in ethical, legal, and societal implications (ELSI) of human genomics research [24], NCGENES2 [25], and CARTaGENE project [26, 27].	Public understanding of precision public health, genetic and genomic risk communication, adequate reach of precision public health interventions, precision public health intervention development and testing of interventions to promote behavior change, and new behavioral targets that may be informed by precision-based approaches	• **Health Policy:** Supporting implementation of cascade screening; ethical implementation of new human genomics discoveries. • **Health Equity:** conduct subgroup analyses to examine how different dimensions of social position interact to shape vulnerability to behavioral risk factors for poor health.

*Biostatistics* involves the study of theories and techniques for collecting, analyzing, and interpreting quantitative data relevant to public health [16]. The immense size and scale of the datasets used in precision public health research necessitate advanced statistical methods with enhanced approaches for reducing noise and enhancing signal detection. These datasets often include multidimensional genetic biomarker data from large numbers of subjects with serial measures repeated over time [28]. Indeed, current genomic technologies allow for the entire human genome to be sequenced through whole genome sequencing. Results from the 1000 Genomes Project suggest that a typical human genome differs from the reference at 4.1 to 5 million sites [17]. In addition to these genomic biomarkers, the ability to detect other biomarkers including the transcriptome, proteome, and metabolome is also increasing significantly. Taken together, the scale of the individual biomarker data that is critical to advancing precision public health, in addition to the complexity of modeling biologic, behavioral, and environmental factors, is mathematically challenging. Thus, the development of advanced statistical methods that allow estimation of interactions between individual-level (e.g., genetic, behavioral factors) and macro-level factors (e.g., environmental exposures and policies) and to identify latent relationships using artificial intelligence (AI) will be necessary.

One example of existing precision public health initiatives within the biostatistics domain of public health is the Genetic Analysis Workshop [18]. This is a collaboration of genetic epidemiologists and statisticians who evaluate and compare statistical genetic methods for multi-omics data including evaluations of causal modeling for multi-omics data and of quality control of high-throughput methylation data for epigenetics studies. These efforts to improve data and modeling quality may help to optimize algorithms for decision support in disease prevention and treatment contexts. Specific examples of this can already be seen for genomics with the ASK2ME (All Syndromes Known to Man Evaluator), which calculates risk of cancers associated with gene variants using data from published studies [19]. Further development of these types of methods and integration with technology, such as the development of algorithms for decision support tools and data resources using AI, can improve risk-stratified cancer prevention and control efforts which are critical to advancing precision public health.

*Environmental health* focuses on the interplay between individuals and their environment to promote health. The field of environmental health has long noted that individual-level factors, such as genetics, interact with macro-level environmental factors to impact population health. The integration of human genomic research within environmental health research may help to elucidate how individual-level factors (e.g., -omics data) and macro-level factors interact to influence health, providing a better understanding of mechanisms underlying complex gene-environment interactions. Understanding how the “exposome” impacts disease development and progression will allow precision public health researchers to target and refine interventions for populations at high risk of poor health outcomes. For example, a gene-environment interaction study using international cohort data assessed whether the association between traffic-related air pollution exposure and incident childhood asthma was associated with genetic factors. Results identified an interaction with air pollution for three single-nucleotide polymorphisms, suggesting that gene-environment interactions may be associated with asthma development [29]. Individuals with these known polymorphisms could be advised to limit time spent outdoors on days where air pollution is particularly high, a development that is already underway within smart city-enabled built environment initiatives [30]. Other work has identified potential gene-environment interactions between workplace exposures to asbestos and family history of mesothelioma [31]; given increased risk of hazardous workplace exposures among underserved populations, this may have implications for our understanding of and interventions related to reducing socioeconomic disparities in health outcomes [32]. In another example, investigators from the Parkinson’s Genes and Environment Study collected mobile health technology data, as well as environmental, genetic, and personal health information longitudinally in order to better understand the link between pesticide exposure, genetics, and Parkinson’s disease [20, 21]. Future work to understand mechanisms behind complex environmental exposures and their interactions with individual-level factors may lead to environmental interventions that target populations at high risk of adverse outcomes [33, 34].

*Epidemiology* uses analytic methods to assess patterns and causes of health states in populations, which encompass individual-level characteristics (e.g., genetics) as well as macro-level factors (e.g., neighborhood and community characteristics)*.* Precision public health draws on epidemiological methods such as population-level surveillance to improve knowledge of multi-level health risk factors which can inform risk-stratified screening, treatment, and prevention interventions. Given advances in human genomic technologies, it is anticipated that genomic information (along with other individual risk factors) will be incorporated into precision public health strategies [33, 35, 36]. These strategies, informed by genetic epidemiological studies, will be critical to promoting positive health-related practices [37, 38], risk-stratified screening and treatment, reducing overdiagnosis, and developing more personalized interventions [39–41]. To this end, examples of human genomics research in epidemiology include investigating associations between multiple common gene variants with disease risk [35, 42, 43], the relationship between genomic (polygenic) variants and monogenic variants [44], the contribution of common variants to risk prediction models along with other risk factors (potentially including other genetic risk information, such as higher penetrance single genes) [45, 46], the role of genetics in identifying rare diseases in newborn screening [4], the epigenome [47, 48], and the interplay between biomarkers and drug response across diverse communities [49–51]. For example, the Northwest-Alaska Pharmacogenetics Research Network is partnering with American Indian and Alaska Native communities to identify known and novel genetic variants associated with drug response and health status in their communities (vitamin D levels, vitamin K nutrition sources, and metabolism related to blood clotting) [22]. Including racially/ethnically diverse populations in human genomic research, such as this one, will contribute to a more generalizable evidence base to improve equity of resulting precision public health interventions [22]. In addition to understanding drug response, evidence from another study that incorporated genetic information into a risk prediction algorithm suggests that some women may benefit from earlier than recommended breast cancer screening, while other women may not be harmed by delaying the start of routine screening [52]. Continued research examining genetic interactions with contextual-level factors will inform more precise prevention and screening which target those who stand to benefit the most from these strategies [53].

*Health policy and health services research* examines quality, cost, and access to health care among the population. These data are essential to understand the economic and health impact of precision public health initiatives, including whether they support health equity. With the evolution of genomic testing in both treatment and prevention settings, health services research will be key in the evaluation of clinical utility, cost-effectiveness, and patient-reported outcomes. These data will be essential in building the evidence base needed for policymakers and practitioners alike to translate human genomic research in precision public health to benefit populations. In particular, the field of implementation science has the potential to play a pivotal role in leveraging precision medicine to benefit population health and to promote health equity (i.e., enabling precision public health) [54–56]. Implementation science is the study of strategies to adopt and integrate evidence-based health interventions such as those generated within the field of genomic medicine into clinical and community settings to improve individual outcomes and benefit population health [57]. Currently, the Implementing Genomic in Practice Consortium [23], funded by the National Human Genome Research Institute, is seeking to enhance the use of genomic medicine in part by integrating implementation science frameworks into delivery of precision medicine. These efforts have resulted in the development of implementation guides for health care systems, clinics, and provider champions who are interested in implementing pharmacogenomics testing [58, 59]. More specifically, the Consortium has developed guidance for implementing and maintaining CYP2D6 testing to guide opioid prescribing as well as CYP2C19 testing for clopidogrel prescription [58]. Across the world, implementation science is at the forefront of human genomics programs and precision public health initiatives, a further example being the Australian Genomics Health Alliance. This national partnership aims to assess the integration of human genomics into the public healthcare system, using implementation science to guide and evaluate the various efforts across more than 80 organizations [60]. Research by this Alliance studying the implementation of ultra-rapid exome sequencing in the neonatal or pediatric intensive care setting has highlighted the complexity of healthcare systems and the necessity of adapting to local context, fostering participatory culture, collective learning, and distributed leadership [61].

*Social and behavioral research* studies ways that behaviors and the conditions of daily life (i.e., the social determinants of health) shape health across the life course [62]. Historically, social and behavioral research related to precision public health has focused on ethical, legal, and social implications (ELSI) of human genomics research [24]. Given the more traditional understanding of genetics as being familial, social and behavioral research has contributed to understanding the ELSI of sharing genetic information with family members by exploring concepts such as autonomy and beneficence. Numerous calls have also been made for social and behavioral researchers to go beyond impacting human genomics research by integrating human genomics considerations within precision public health initiatives [63, 64]. Examples of current and future precision public health research opportunities in the social and behavioral sciences include: improving public understanding of genomics and data sharing, genetic and genomic risk communication, expanding the reach of precision public health interventions, developing and testing precision public health interventions to promote behavior change, and identifying new and/or more precise behavioral targets that may be informed by precision public health approaches [65]. Research in this area promises to improve public trust and willingness to participate in interventions that address their genetic risks.

To date, social and behavioral scientists have made progress in translating genomic applications for the benefit of population health by using community-based genomic research that includes communities as partners in human genomic research [53, 66]. For example, the North Carolina Clinical Genomic Evaluation by Next-generation Exome Sequencing 2 (NCGENES2), a diagnostic genomic sequencing study for children with undiagnosed conditions, explored the impact of a Community Consult Team consisting of clinicians and caregivers in order to maximize the utility and equity of genomic sequencing [25]. Engaging this team resulted in adaptations to the study protocol and materials, demonstrating the valuable contribution of community engagement to inform the implementation of translational human genomics research [67]. Another example, the Quebec CARTaGENE project, a large-scale genetic database that serves as a public research platform, engaged the public about their willingness to participate in such a project. The first public engagement highlighted the importance of transparency, maintaining confidentiality, and ensuring the ability for donors to provide feedback and guidance. Further, the team found a need to tailor and target communication about the project across patient populations [26, 27]. Other related research has incorporated the patient voice by assessing patient and family preferences for learning genomics information in order to inform family-based interventions that aim to disseminate genetic information to families at high risk of disease [68–72].

## Cross-cutting challenges and opportunities for integrating human genomics research within precision public health

As demonstrated by examples from the field, principles of precision public health are being applied by researchers working at the intersections of precision medicine, genomics, and public health. In addition to domain-specific advances, innovations cutting across all domains are needed to advance precision public health initiatives, including (1) revised health policies, (2) improved data integration and development of more rigorous methodologies, and (3) an emphasis on health equity (Table 1) [73–75].

*Health policies* at all levels including clinic, industry, health system, state, and federal will need to adapt to rapidly growing precision-based approaches in order to support the goals of precision public health.

The development of new policies focused on ensuring the privacy, confidentiality, and regulation of genetic results delivered to individuals will be important as precision public health becomes more established due to the potential for precision health data to be highly identifiable. Further, as our ability to predict public health outcomes improves, we must consider when and how to intervene and the ethical and legal ramifications of action or inaction. For example, researchers have identified a link between child abuse and antisocial behavior with a common monoamine oxidase (MAOA) gene variant [76]. A meta-analysis of 27 studies identified a robust association between increased likelihood of antisocial outcomes after childhood adversity among carriers of the low-activity MAOA genotype. Definitions of childhood adversity and antisocial behavior varied, with the strongest association with childhood maltreatment. However, an intervention based on these data, presumably to reduce child abuse, should be a universal goal and not targeted towards a specific subpopulation with low-activity MAOA genotype. With each new epidemiological association, policymakers must decide whether and how the results should be used to advance precision public health. Policies that are proactive in addressing the changing landscape will be essential, as rapid advances in precision public health become available. Experience with ELSI could help inform decision making about these new discoveries and how they can be ethically implemented and translated into new policies. Specifically, there are a number of Centers of Excellence in ELSI Research (CEER) funded by the National Human Genome Research Institute. These projects use interdisciplinary approaches to address complex and rapidly emerging ethical, legal, and societal issues that occur as research advances in genomics [77].

In addition, certain limitations in current policies must be addressed. For example, in the USA, the Genetic Information Non-Discrimination Act fails to protect the public from life insurance discrimination based on pre-existing genetic conditions, most greatly impacting socioeconomically vulnerable citizens who may not be able to afford life insurance rates that are raised due to their genetic risks [78]. Conversely, US state-level genetic privacy laws may inhibit the sharing of important genetic information with relatives, and while this may be important in protecting genetic privacy, it may also complicate precision public health efforts to implement cascade screening (or family genetic testing) to identify and manage disease risk among individuals with highly penetrant genetic conditions [79]. Future research studying the impact of current policies, as well as barriers and facilitators to enacting these policies, will be essential for building a policy landscape that facilitates precision public health.

*Improved data integration and methods* will be necessary to leverage the complexity of human genomics data for addressing precision public health problems. Incorporating these data with other individual-, interpersonal-, community-, and environmental-level data in a meaningful and rigorous way will require novel methods for data measurement, collection, management, and integration [73]. In particular, advanced informatics is needed to fulfill the opportunities and meet the challenges of integrating human genomic research within precision public health through information technology infrastructure development [74]. As the underpinnings of multi-level mechanisms take shape, multi-level systems-based interventions will be necessary. These include study designs and data storage infrastructure that allow researchers to understand both the synergistic and independent effects of these complex problems and multi-level solutions across diverse populations. Future opportunities in this area include improving complex study designs and big data management and storage. In addition, researchers will need secure data systems that allow data to be shared efficiently and ethically between researchers and public health agencies and practitioners. Further, data integration of genomic and other precision public health data into the electronic health record with appropriate decision support will be important in integrating these data into the clinical setting to improve health [75, 80, 81].

One application of improved data integration and related methods to help address precision public health problems is through the use of conditional random forests (a machine learning approach). Nau et al. used this approach to identify features of social, food, and physical activity environments that jointly predicted whether a community’s environment could be considered as primarily obesogenic or obesoprotective [82]. The authors used a dichotomous indicator of average BMI *z*-score among children living in each community as a proxy for each of these outcomes. Of the 44 community characteristics examined, a combination of 13 features of the social, food, and physical activity environment correctly classified two-thirds of communities as obesogenic or obesoprotective, with social features being the most informative in this respect. This study offers a way forward in use of large datasets to help define more precise targets for public health intervention, which are critical to precision public health initiatives.

*Health equity*, or fair access to resources that allow for an individual to reach their full health potential, is integral to the future of precision public health. Broadly, ensuring health equity requires that individuals have access to appropriate resources regardless of their social position or circumstances. As it relates to precision public health, Olstad and McIntyre specifically define “precision public health” as “the study of how multiple dimensions of social position interact to confer health risk differently for precisely defined population subgroups according to the social contexts in which they are embedded, while considering relevant biological and behavioral factors” [7]. Enhancing attention to health equity is perhaps the most important opportunity for the intersection of precision- and population-based approaches if we are to ensure that precision public health retains its focus on improving health for all members of society, and the health of marginalized groups in particular [53, 83].

Unfortunately, racial, ethnic, and socioeconomic disparities in access to precision approaches, including genetic testing, have already emerged [84, 85]. For example , underrepresentation of racial and ethnic minority populations in genomic databases limits the benefit of newly developed tools for assessing risk, prognosis, and therapeutic response for patients who already experience limited access to and poorer quality of healthcare due to factors such as poverty and systemic racism [86]. Specific efforts have been taken to implement genomic screening for hereditary breast and ovarian cancer syndrome (HBOC), Lynch syndrome (LS), and familial hypercholesterolemia (FH) among diverse populations (i.e., non-European ancestry populations) that are commonly excluded in genomic medicine research [87]. Most participants in this diverse sample opted for the return of results, with younger participants, female participants, and Hispanic/Latinx participants being most likely to opt to receive their results [88].

Community engagement, a cornerstone of public health research, may facilitate the implementation of precision public health initiatives seeking to promote health equity [10]. Indeed, initiatives such as the All of Us Research Program have recognized a need to restore trust among underserved and historically marginalized groups, such as Black, LatinX, and Indigenous American groups, to participate in health research through community engagement principles [89, 90]. Thus, it is critical to ensure that equity remains a core concern within precision public health initiatives [91]. With its emphasis on health equity and incorporating multiple levels of influence on population health, human genomic research can play a major role in precision public health research that impacts population health [92]. This can be achieved through increasing inclusion of underrepresented populations in genomics research to ensure that all of society shares in the economic and health benefits of precision public health investments [93].

## Conclusions

While challenges lie ahead, the increased integration of human genomics research in precision public health is expected to provide opportunities for improving population health. To this end, there is a need to consider the most acceptable and effective way to scale advances in human genomic research to benefit populations by better targeting disease prevention, treatment, and control efforts. Transdisciplinary research that draws from precision medicine, genomics, and public health can continue to serve as a needed conduit towards the realization of the full range of opportunities in the emerging field of precision public health.

## Data Availability

Not applicable
